# Lancet Migration European Regional Hub: Working together towards inclusive, evidence-based outcomes for promoting and protecting the health of migrants in the European Region

**DOI:** 10.1016/j.lanepe.2022.100534

**Published:** 2022-10-29

**Authors:** Rosemary James, Karl Blanchet, Bernadette Nirmal Kumar

**Affiliations:** a Lancet Migration European Regional Hub, Switzerland; b Geneva Centre of Humanitarian Studies, Geneva, Switzerland; c Norwegian Institute of Public Health, Oslo, Norway

Migration and health, a defining issue of our times, has increasingly gained importance in Europe. The COVID-19 pandemic and war in Ukraine have further highlighted the need for a strong community of researchers and health professionals to focus their efforts on documenting the inequities, needs and aspirations of migrants and to explore solutions to these.^1^^,^^2^ The Lancet Migration European Regional Hub has emerged as a catalyst for collaboration to harness cross-country synergies and drive evidence-based policy.

The Hub's objectives are fourfold: (i) to facilitate evidence generation across the European region through intercountry collaboration; (ii) to disseminate evidence and promote best practices to stakeholders in migration and health; (iii) to act as a nexus of expertise supporting research, practice, and policy dialogue within the region; and (iv) to enable the next generation of migration and health researchers in their learning and career pathways.

Since the Hub's inception in January 2021,^3^ it was time in July 2022 to convene a strategic workshop in Geneva, with sixteen regional migration health experts to discuss the Hub's progress to date, and to determine key priorities for research collaboration in the European Region. During this workshop, challenges facing migrant health professionals and researchers were discussed, as well as possible strategies to address them. Multiple gaps in evidence and research methods were further uncovered. The group will have three working groups for the next two years: (i) Universal Health Coverage (UHC); (ii) Vaccination coverage with a special focus on COVID-19; (iii) and Border Security, detention and health (Table 1).

**Table 1:** Current thematic priorities for the Lancet Migration European Regional Hub.

Theme	Key priority areas	Cross-cutting priorities
Universal Health Coverage and migration	• Identification of innovative approaches, models, and opportunities to advance a more migrant-inclusive UHC at regional, national, and local levels	• Establishing an evidence base of inclusive policies and good practices • Assessment of gaps in policies and service delivery • Reviewing and evolving research methodology • Targeted dissemination of evidence
Vaccination coverage with a special focus on COVID-19	• Defining innovative models of care and good practices for improving vaccination coverage • Drive momentum from COVID-19 vaccination toward more inclusive vaccination programmes for migrants in Europe	
Border security, detention, and health	• Revising and examining key principles of European migration policy frameworks and their implication on the health of people migrating as well on practices and ethics of health practitioners	

Achievement of UHC is dependent on cross-sectoral collaborations.^4^^,^^5^ Workshop participants raised concerns about undocumented migrants being excluded from basic health service provision, especially in areas with border or detention zones that often lack public health interventions and primary healthcare facilities. In particular, chronic diseases require more attention, across the life course. Health service delivery needs to be migrant-inclusive and culturally sensitive, especially for mental health. Urgent action is needed to support the World Health Organization's (WHO) Special Initiative for Mental Health, which is in line with UHC.^6^

Undocumented migrants continue to be excluded or not caught-up in immunization programmes across Europe.^7^ This is due not only to restrictive policies, but a multitude of factors including healthcare provider awareness, public communication, and health literacy.

A significant barrier to studying and improving the health of migrants in European countries has been the difficulty in collecting and accessing data in detainment settings particularly due to restrictions on people's movements and access to care. The mainstream narrative surrounding migration to and within Europe has often been fuelled by politics and has stigmatized migrants as a threat,^8^^,^^9^ although evidence demonstrates the contrary.^1^^,^^2^^,^^7^ Amidst a new era of policies such as the New Pact on Migration and Asylum proposed by the European Commission,^10^ and concerns over recent plans by the United Kingdom to deport asylum applicants abroad, it is critical to generate cross-border evidence that documents the consequences of such policies health and wellbeing.

There is a need to ensure basic data on migrants is included in routine national data collection, and to disaggregate data by migrant status and other demographic indicators, to identify inequities between groups. The European region has over twenty official languages. Translation and sharing of best practices and tools will help to create cross-border synergies. Migrants need to be placed at the centre of health services and health research through participatory approaches. Current narratives, misconceptions, and stereotypes towards all types of migrants need to be addressed, through evidence-based dialogue.

The Hub's network of experts will support countries to address the above issues. Country focal points and special advisors for migration and health research will be identified, and collaborations with other networks within and outside Europe will be established. Over time, the Hub will aim to serve as a trusted ‘Think Tank’ for migration and health research in the European region.

In 2023, a series focusing on ‘Migration and Health Inequities’ will be published in collaboration with *The Lancet Regional Health - Europe*. This series aims to build on the 2018 UCL-*Lancet* Commission on Migration and Health recommendations and provide the European regional perspective five years after the publication of the Commission report.^11^ Specifically, the series will address overarching thematic issues as well as update some key areas of interest regarding migrant health with the latest research developments. Lessons learned, particularly surrounding equity and migrant-inclusive health systems, including the wider determinants of health, will be showcased. Evidence from different countries and disciplines will be synthesised to inform policy. There will be a range of articles co-authored by some of the best experts in the region.

To read more about the Lancet Migration European Regional Hub, visit migrationhealth.org/regional-hubs/europe.

## Contributors

BK and KB conceptualised the piece. RJ and KB drafted the initial comment. KB, BK, and RJ provided revisions.

## Declaration of interests

There are no conflicts of interests to declare.
